# Developing and testing personalised nutrition feedback for more sustainable healthy diets: the MyPlanetDiet randomised controlled trial protocol

**DOI:** 10.1007/s00394-024-03457-0

**Published:** 2024-07-06

**Authors:** Katie P. Davies, Eileen R. Gibney, Ursula M. Leonard, Leona Lindberg, Jayne V. Woodside, Mairead E. Kiely, Anne P. Nugent, Elena Arranz, Marie C. Conway, Sinead N. McCarthy, Aifric M. O’Sullivan

**Affiliations:** 1 UCD Institute of Food and Health, School of Agriculture and Food Science, University College Dublin, Dublin, Ireland; 2 Cork Centre for Vitamin D and Nutrition Research, School of Food and Nutritional Sciences, University College Cork, Cork, Ireland; 3 Centre for Public Health, School of Medicine, Dentistry and Biomedical Sciences, Queen’s University Belfast, Northern Ireland, BT12 6BJ, Belfast, UK; 4 Institute for Global Food Security, School of Biological Sciences, Queen’s University Belfast, Belfast, BT9 5DL UK; 5 Department of Nutrition and Food Science, Complutense University of Madrid (UCM), Madrid, Spain; 6 Department of Agrifood Business and Spatial Analysis, Teagasc Food Research Centre, Ashtown, Dublin, Ireland

**Keywords:** Sustainable diets, Personalised nutrition, Randomised controlled trial

## Abstract

**Purpose:**

Agriculture and food production contribute to climate change. There is mounting pressure to transition to diets with less environmental impact while maintaining nutritional adequacy. MyPlanetDiet aimed to reduce diet-related greenhouse gas emissions (GHGE) in a safe, nutritionally adequate, and acceptable manner. This paper describes the trial protocol, development, and testing of personalised nutrition feedback in the MyPlanetDiet randomised controlled trial (RCT).

**Methods:**

MyPlanetDiet was a 12-week RCT that provided standardised personalised nutrition feedback to participants based on new sustainable healthy eating guidelines (intervention) or existing healthy eating guidelines (control) using decision trees and corresponding feedback messages. To test the personalised nutrition feedback, we modelled a sample of 20 of the MyPlanetDiet participants baseline diets. Diets were modelled to adhere to control and intervention decision trees and feedback messages. Modelled nutrient intakes and environmental metrics were compared using repeated measure one-way analysis of covariance.

**Results:**

Intervention diets had significantly lower (*p* < 0.001) diet-related GHGE per 2500 kilocalories (kcal) (4.7 kg CO_2_-eq) relative to control (6.6 kg CO_2_-eq) and baseline (7.1 kg CO_2_-eq). Modelled control and intervention diets had higher mean daily intakes of macronutrients (carbohydrates, fibre, and protein) and micronutrients (calcium, iron, zinc, and iodine). Modelled control and intervention diets had lower percent energy from fat and saturated fat relative to baseline.

**Conclusions:**

Adherence to the MyPlanetDiet personalised nutrition feedback would be expected to lead to better nutrient intakes and reduced diet-related GHGE. The MyPlanetDiet RCT will test the effectiveness and safety of personalised feedback for a more sustainable diet.

**Trial registration number and date of registration:**

Clinical trials registration number: NCT05253547, 23 February 2022

**Supplementary Information:**

The online version contains supplementary material available at 10.1007/s00394-024-03457-0.

## Introduction

Agriculture and food production play a critical role in mitigating climate change and meeting global climate targets [1–3]. Research shows that changing dietary behaviour can reduce diet-related environmental impact indicators, like greenhouse gas emissions (GHGE) [3–7]. Sustainable healthy diets that have lower environmental impact are generally rich in plant-based foods, such as whole grains, fruits and vegetables, legumes and nuts and seeds, with low to moderate amounts of animal-sourced foods [8]. Most food-based dietary guidelines recommend a similar dietary pattern but provide vague and generic eating advice using broad terms like ‘increase’ or ‘avoid’ [8, 9]. Other recommendations for sustainable diets with specific intake recommendations (grams per day) have been published but have been called into question by others as to whether these diets are nutritionally adequate, or culturally acceptable [3, 6, 7, 10–14].

To date, the evidence base for sustainable diets has been mostly theoretical and often examined using modelled dietary changes. For example, in a systematic review by Aleksandrowicz and colleagues where different dietary patterns were modelled to examine the impact of dietary change on environmental metrics [4]. Several research groups have published ‘reference diets’ which serve as a model for a sustainable diet [3, 6, 7]. These diets provide an example diet with specific intake recommendations for key food groups but have yet to be expanded to account for the variability in nutrition needs across the population. Factors such as demographics (e.g. age, sex, body size), behaviour (e.g. dietary intake, physical activity), and genetics influence an individual’s nutrition needs [15]. Using these factors to provide more tailored or targeted nutrition advice is known as personalised nutrition. Research shows that personalised nutrition advice leads to healthier eating behaviour over a longer period when compared to a traditional, ‘one-size-fits-all’ approach [16–18]. The multi-national Food4Me study compared multiple levels of personalised nutrition feedback to general healthy eating advice and showed that personalised nutrition advice can be delivered using a standardised decision-making framework that is scalable and reproducible [19]. The Food4Me study also showed that the messages derived from decision trees aligned with personalised advice from a dietitian and concluded the decision tree method could be effective in large populations due to its efficiency and reliability [20]. To our knowledge, personalised nutrition has yet to be tested in relation to achieving more sustainable healthy diets. Similarly, there is a research gap with respect to the effectiveness of studies providing dietary advice aimed at reducing diet-related environmental impact.

The aim of this paper is to describe the development, testing, and use of a novel personalised feedback framework designed to deliver dietary advice to reduce diet-related GHGE in a healthy and acceptable manner as part of the MyPlanetDiet RCT. This paper will provide an overview of the MyPlanetDiet dietary protocol, including the development and testing of decision trees and feedback messages used to deliver personalised dietary advice to participants.

## Methods

### Myplanetdiet study design

MyPlanetDiet was a 12-week single-blinded parallel RCT evaluating the capacity for a more sustainable diet to reduce diet-related GHGE in a healthy, acceptable, and safe manner. Participants were randomized into one of two groups of personalised nutrition feedback based on either a more sustainable and healthy diet (intervention) or healthy eating food-based dietary guidelines, from the Republic of Ireland and Northern Ireland (control) [21, 22]. Participants in the two groups received the same level, type, and frequency of communication with the only difference being the target diet that underpinned the advice provided to both groups.

MyPlanetDiet included two onsite visits per participant (visit 1/week 0 and visit 2/week 12). The primary outcome was change in diet-related GHGEs from baseline to endpoint. Secondary outcomes included changes in nutrient intake (energy, macronutrients, vitamins, and minerals), nutritional status (blood and urine biomarkers), health status (body weight, body mass index [BMI], waist circumference, blood pressure, metabolic function, clinical chemistry, gut microbiota composition), acceptability of the dietary changes and additional environmental indicators.

### Study participants and recruitment

The Human Research Ethics Committee in University College Dublin (LS-21-51-Davies-OSullivan) (affirmed by Faculty of Medicine, Health and Life Sciences Research Ethics Committee, Queen’s University Belfast MHLS_21_109) and the Clinical Research Ethics Committee of the Cork Teaching Hospitals in University College Cork (ECM 4 (cc) 10/8/2021 & ECM 3 (f) 19/10/2021) granted ethical approval for the study. Prior to beginning the study, participants received a Participation Information Leaflet and asked to sign consent (Appendix A). The study was carried out in line with the principles set forth in the Declaration of Helsinki. Participants were able to discontinue at any time at which point no further data was collected. Participants also had the option to withdraw their consent at which point data collected from the individual was destroyed. A standard operating procedure (SOP) was developed and agreed upon by all study site investigators. The SOP (version 1.0, 29 March 2022) for MyPlanetDiet is described here using SPIRIT reporting guidelines [23]. The protocol included procedures for conducting the trial and how to manage deviations. Participants who deviated from protocol continued the trial as close to protocol as possible and deviations were noted in participant logs.

The study aimed to recruit 360 participants evenly across three universities: University College Dublin, University College Cork, and Queen’s University Belfast. Participants were recruited through advertisements via email, radio, posters, public speaking events and social media. Sample size (80% power, 5% significance) was calculated based on achieving a 20% difference in GHGE between intervention and control diets. Allowing for potential age and sex differences and a 25% potential dropout rate, a total of 360 participants was required across the three study sites.

Healthy adults aged 18–64 years who consumed a moderate-to-high GHGE diet (self-reported red meat intake of ≥ 3 portions per week) were eligible to take part. Screening questions included demographic characteristics (e.g. age, sex) and lifestyle behaviours, including questions relating to habitual intake of critical food groups such as red meat, white meat, fish, eggs, and plant protein. The following exclusion criteria were applied;

- Pregnancy, currently breastfeeding or females planning to become pregnant.
- Following a medically prescribed diet.
- Diagnosis of an acute or chronic condition that may interfere with the outcomes of the study. Conditions that are excluded include (but are not limited to) diabetes mellitus, inflammatory bowel disease, recent history/ongoing cancer treatment.
- Immunocompromised or have a suspected immunodeficiency.
- Excessive alcohol intake (> 28 units of alcohol consumed per week).
- Known food allergies.
- Regular consumption of a single high-dose vitamin or mineral supplement.
- Participation in another research study.
- Inability to read, write or understand English.

### Randomisation and blinding

Following consent, participants were stratified by sex (female or male) and age (≤ 40, > 40 years), and randomised using site-specific blocked randomisation list to the intervention or control. As each new participant was recruited, they were allocated to the next available and ID code based on respective sex and age. Participants were blinded to their study; however, researchers were not.

### Dietary intake and analysis

Dietary intake was measured prior to commencing the study or attending visit 1 (referred to from this point onwards as the “baseline” dietary assessment) with follow-ups at week 6 and week 12 (Table 1). Participants completed 3-online 24-hour recalls on non-consecutive days and a food frequency questionnaire at each timepoint using Foodbook24, a validated online dietary assessment tool [24]. Dietary intake data from the three 24-hour recalls was downloaded directly from Foodbook24 and includes nutrient composition as previously described [25]. Food frequency data were not used for the present analysis. Participants enrolled in MyPlanetDiet received personalised nutrition feedback based on mean daily intakes of nutrients and key food groups (as detailed below). A database was created to standardise how foods in the Foodbook24 food list contribute to relevant food groups. Single foods were directly matched with the relevant food group. Composite dishes were disaggregated to food group level using 3–5 online recipes. The mean ingredient amount (g) per 100 g of recipe was calculated accounting for cooking factors derived by McCance and Widdowson Composition of Food Integrated Dataset [26]. Ingredients weighing less than 10 g of the total recipe weight (< 1%) were excluded. The total weight of the recipe and the total weight of each ingredient were calculated, and the mean of each ingredient of the 3–5 recipes was calculated to show the average contribution per 100 g of recipe. Each ingredient was matched with a relevant food group; for example, a cheeseburger will contribute to red meat, dairy, and vegetable food groups.

### Diet-related environmental impact data

All foods in the Foodbook24 database were assigned GHGE and water footprint values per 100 g of food using life cycle assessment data (LCA) published by Colombo and colleagues [27] in the UK, taking account of the proportion of local production and imported foods which is similar in Ireland [27, 28]. The environmental data published by Colombo and colleagues included LCA data from previous studies which encompass over 50 LCAs [27]. Colombo and colleagues further refined the environmental database to include 266 foods or food groups [27]. A stepwise procedure mapped foods from the Foodbook24 database to environmental data, starting with foods that mapped directly. The next step mapped composite dishes to their relevant food groups using the recipe database described in the previous section. The final step took foods that did not have respective environmental values and allocated them to a similar food group. The data was quality controlled, and a syntax was created to connect the food consumption database with the environmental impact database.

### Sample and data collection

Participant data was collected at five timepoints (Table 1). Following screening and consent, participants completed a health and lifestyle questionnaire and the first dietary assessment (3-online 24-hour recalls and 1 food frequency questionnaire) at baseline prior to attending visit 1. The health and lifestyle questionnaire included socio-demographics, health behaviours, and self-reported anthropometrics which was used to estimate energy (kcal) requirements for the individual [29]. Participants were invited to attend two onsite visits at a study centre at the start of week 0 (visit 1) and at the end of week 12 (visit 2). At each visit, fasting anthropometry (height, body composition, hip circumference, waist circumference), clinical measurements (blood pressure) and biological samples (blood and urine samples and optional faecal samples) were collected. Biological samples will be used to measure changes in metabolic health, nutrient status, and gut microbiome composition. More detail on biological sample collect and data analysis can be found in Appendix B. All data was collected using standard operating procedures. Participants completed questionnaires onsite during visits including food waste, stage of change and diet-change tolerability using Qualtrics (Qualtrics XM, Seattle, USA), an online survey tool. The 36-item food waste questionnaire was developed and validated by Stancu and colleagues and included self-reported food waste quantities, behaviours associated with food waste, and attitudes towards food waste [30]. Participants completed a stage of change questionnaire at visit 1 which used an algorithm previously used in dietary change studies to assess readiness for dietary change [31]. Participants completed a tolerability questionnaire (adapted from a previous dietary intervention) at visit 2 to test the acceptability of the dietary changes, general well-being of participants, and ease of dietary changes [32].

**Table 1:** MyPlanetDiet data and sample collection

Outcome	Time Point				
	Screening	Baseline	Visit 1 (Week 0)	Week 6	Visit 2 (Week 12)
Demographics	**√**	**√**			
Eligibility	**√**				
Consent		**√**	**√**		
Health and lifestyle		**√**			
Foodbook24		**√**		**√**	**√**
Blood sample			**√**		**√**
Urine sample			**√**		**√**
Faecal samples			**√**		**√**
Anthropometry			**√**		**√**
Food waste			**√**		**√**
Stage of change			**√**		
Diet-change tolerability					**√**

### Participant communication

All study resources, participant communication guides and nutritionist training were designed to ensure consistent participant interaction and engagement across groups and study sites. MyPlanetDiet participants were blinded, therefore no feedback message included language related to sustainability. A trained study nutritionist discussed the personalised nutrition feedback with each participant in-person using a feedback report (described later) at onsite visit 1 (week 0) and followed up with participants at weeks 1, 3, 6 and 9 to check-in on progress and reiterate dietary targets and improve adherence to study protocols.

### Data management and analysis

An independent central data monitor was appointed from the project consortium. A data management plan was developed and agreed by all partners which included plans for data monitoring, sharing and dissemination. Upon signing informed consent, participants were allocated a study code which was used to store participant data during the trial. A file linking a participant’s information to their study code was stored during the trial in site-specific password protected files that only named researchers had access to. Data was deidentified upon study completion. Only project researchers will have access to datasets until grant completion. Dietary assessment and questionnaire data were exported from Foodbook24 and Qualtrics. Anthropometry data was input by researchers from case report forms. Data dictionaries were created and standardised across sites to code data. Deidentified data was merged across sites and quality controlled by researchers. Upon conclusion of the study, identifiable data was destroyed in accordance with relevant data protection acts. Biological samples will be destroyed after ten years to comply with the study’s ethical approval. Primary and secondary outcomes such as diet-related GHGEs, nutrient intake, nutrition status, and health status will be compared between intervention and control groups using general linear model (GLM) two-way repeated measures analysis of covariance (ANCOVA), controlling for covariates such as sex, age, BMI, and energy intake. Pearson’s correlation will be used to determine possible covariates. Results will be disseminated through academic journals, conference presentations and public speaking engagements.

### Personalised nutrition feedback

#### Development of personalised nutrition feedback

**Fig. 1:**
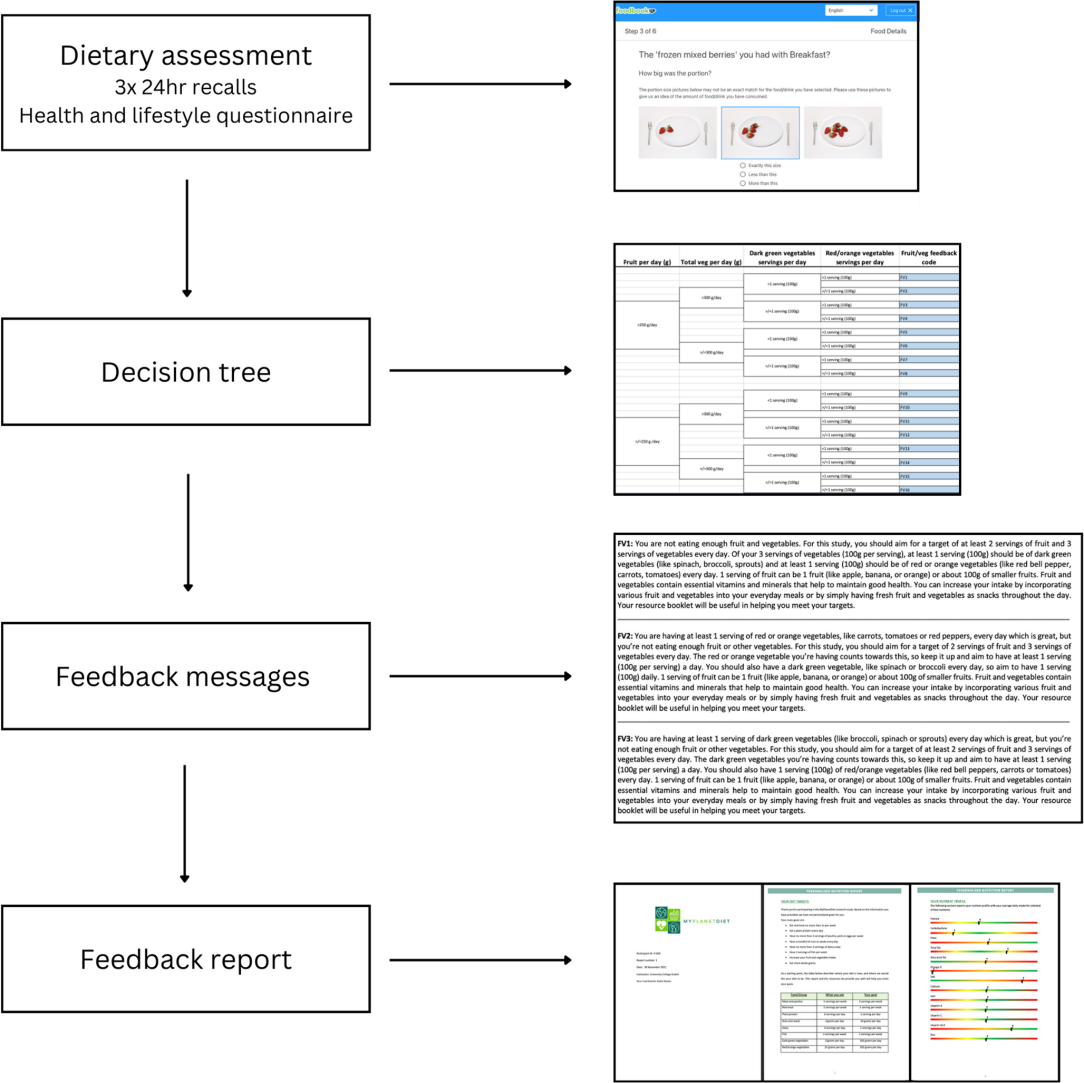
Stages of delivering personalised feedback

The process of delivering personalised nutrition feedback included 4 stages: assessing dietary intake, using decision trees, selecting feedback messages, and compiling a feedback report (Fig. 1). This standardised process has been previously used and described in more detail [19, 24]. An individual’s dietary intake can be assessed based on their intake of critical food groups, such as fruit and vegetables, whole grains, dairy and protein foods. Food groups included in the personalised feedback are described in more detail later in this manuscript. Recommended intakes are used in decision trees to create a stepwise process for providing personalised feedback messages. Feedback messages included a specific intake target (e.g. grams per day per food group) and tips for how to achieve their target. For example, if a participant received a message to increase their whole grain intake, they would be provided with suggestions for how to swap refined grains for whole grains in their diet. Feedback messages were compiled into a report and provided to an individual. Feedback reports included 5 sections. ‘Your diet targets’ included individuals’ current intakes (by weight or servings) of key food groups compared to their personalised targets. ‘Your personalised feedback’ had actionable feedback messages derived from decision trees. Messages included specific advice to increase, reduce or maintain intake of food groups. ‘General feedback’ included considerations that could not be specifically personalised such as limiting ‘treat food’ intake. ‘What are your food groups’ included tables to define and explain food groups to the participant. ‘Your nutrient profile’ was a visual representation of baseline intakes, compared to recommended intakes using European Food Safety Authority (EFSA) DRVs [19] for 13 key nutrients and a traffic light system (Fig. 1).

Personalised dietary advice was provided to the intervention and control groups to control for mode of delivery. To develop the personalised dietary feedback for the intervention group, a review of published literature and existing food-based dietary guidelines was conducted to identify critical food groups for a more sustainable diet [33]. Reference diets were compiled and compared to mean daily intakes from the national food consumption survey in Ireland (National Adult Nutrition Survey, NANS) to assess the compatibility between the two [34]. Mean food group intakes and patterns of consumption from NANS informed the personalised feedback messaging. For example, the personalised feedback recommended fewer larger serving sizes of 140–160 g for meat compared to healthy eating guidelines to reflect current dietary patterns. An intervention diet was developed from identified reference diet recommendations, taking into consideration current dietary intakes using data from NANS [3, 6, 7]. Five tiers of dietary advice depending on estimated energy needs (≤ 2249 kcal, 2250-2449 kcal, 2500-2749 kcal, 2750-2999 kcal, ≥ 3000 kcal) were created. Estimated energy needs were calculated by estimating resting metabolic rate with self-reported height and weight using the Mifflin-St Jeor equation and using activity factors which corresponded with self-reported physical activity using the short-form International Physical Activity Questionnaire (IPAQ) [29, 35]. Prior to generating personalised feedback, participants were screened for underreporting and underreporters were given an opportunity to repeat dietary assessments before progressing on the study as was done in the Food4Me study [36]. Dietary advice for each energy group was designed to meet energy needs and maintain similar macronutrient distribution and micronutrient intake. Thirty decision trees were created, five groups of six decision trees separated based on an individual’s daily energy requirement, ranging from 2249 to 3000 + kcal in 250 kcal increments. Decision trees provided feedback on six food groups in the following order: meat, plant protein, fish, dairy, fruit and vegetables, starches. The order of feedback in the intervention was set based on the highest potential impact to reduce diet-related GHGEs.

Personalised nutrition feedback for the control was based on healthy eating guidelines as described previously [24, 37]. Unlike the intervention, the control provided only one tier of recommendations for all energy needs. Decision trees provided feedback on five food groups in the following order: fruit and vegetables, wholegrain, dairy, fish, and red meat. The priority order of the feedback was decided based on the order of advice provided in healthy eating guidelines [21].

The median intake recommendations (grams per day) for intervention and control groups are presented in Table 2. The intervention provided specific recommendations for starchy vegetables, dark green vegetables, red/orange vegetables, and plant proteins.

**Table 2:** Median and range of personalised nutrition feedback recommendations (grams per day) by group

Food group	Intervention	Control
**Breads/cereals/starches**	6 (4–7) servings^a^	--
Whole grains	≥50	≥50
**Tubers/starchy vegetables**	100 (0-150)^b^	--
**Vegetables (all)**	300	240
Dark green	100	--
Red/orange	100	--
Other	100	--
**Fruit**	250 (200–300)	160
**Dairy**	250 (200–300)^c^	350
Cheese	25 (0–25)^b, c^	25
Liquid dairy	250 (175–300)^c^	325
**Animal protein**		
Red meat (beef/lamb)	20 (0–20)^b^	70 (0–70)^b^
Pork	13 (0–15)^b, d^	
Poultry	13 (0–15)^b, d^	--
Eggs	13 (0–15)^b, d^	--
Fish	28 (28–40)	28
**Plant protein**		
Legumes	125 (125–150)	--
Soy and meat alternatives	28 (0–42)^b^	--
Nuts and seeds	30 (25–35)	--

^a^ Serving sizes for breads, cereals, starches were provided to participants

^b^ Recommendations had upper limit (i.e. no more than x grams per day) where 0-upper limit were within recommended range

^c^ Median recommendations for dairy, cheese ,and liquid dairy where intake will depend on optional cheese intake (max 25g per day). Median recommendations included 250g dairy equivalents whereby individuals could have 250g liquid dairy equivalent with 0g cheese or have less liquid dairy and 25g cheese depending on preference

^d^ Pork, poultry and eggs included as one recommendation where an individual could eat any interchangeably

### Decision tree testing

The theoretical effectiveness of the intervention feedback process was assessed by modelling the expected effects on individuals’ diets. A convenience sample (*n* = 20) from the MyPlanetDiet RCT was selected and used to model theoretical outcomes and variation from the control and intervention groups (control and intervention) of the study’s personalised nutrition feedback system. The first five participants who joined the study in each stratification group (females aged 18–40 years, females aged 41–64 years, males aged 18–40 years, males aged 41–64 years) were included in the analysis.

Each participant’s baseline dietary assessments (3x online 24-hour recalls) were entered into Nutritics software (Research Edition, v5.85, Dublin, Ireland). Each participant’s food intake was considered separately and inputted by day (Day 1, Day 2, and Day 3) and sorted by ascending food code. A two-step process was undertaken. Firstly, an individual’s dietary intake was considered within the MyPlanetDiet feedback framework for both intervention and control, resulting in recommended changes to the diet for both groups. In step 2, conditional flow charts were used (Table 3) to adjust baseline diets in accordance with the appropriate feedback messages derived in step 1. Table 3 presents an example of how diets were modelled for fruit and vegetable intake in modelled control diets. Changes were made in order of ascending food code to reduce researcher bias in diet changes. Adjustments were made until the participant’s food intake aligned with feedback messages. Taking the vegetable example presented in Table 3; if a participant consumed 160 g of vegetables, then 80 g of unsalted, boiled mixed vegetables was added to their daily intake to meet the 240 g target. Step 2 was completed for all food groups and repeated for both the intervention and control dietary feedback. Detailed description of dietary modelling protocol is included in Online Resource 1. Following adjustments according to the theoretical changes, the modelled diets were reanalysed using Nutritics. As such, on completion of this exercise, 3 versions of each participant’s diet (Baseline, modelled control, and modelled intervention) were calculated and exported for analysis.

**Table 3:** Example of modelled changes to diets, shown for fruit and vegetables in control group

Food group	Target	Daily intake	Change
Vegetables	240 g	< 240 g	Add difference between 240 g and total daily vegetable intake as mixed vegetables, boiled, unsalted
		≥ 240 g	No change
Citrus fruit	80 g	< 80 g	Add difference between citrus fruit intake and 80 g in as orange
		≥ 80 g	No change
Total fruit	160 g	< 160 g	If below 160 g (including after citrus fruit added in), add in remaining grams as apple
		≥ 160 g	No change
Juice	≤ 125 ml	≤ 125 ml	No change
		> 125 ml	Decrease juice intake to 125 ml

### Statistical analysis

Statistical analysis was performed using IBM SPSS Statistics version 27 (IBM Corp., Armonk, NY, USA). Data are presented as n (%) or means ± SE where appropriate. Variables were checked for normality using Shapiro-Wilk tests and histograms. Mean daily nutrient intakes and environmental indicators were calculated for baseline, modelled control and modelled intervention diets and analysed using GLM repeated measure one-way ANOVA where macro- and micro-nutrient intakes and absolute environmental values were controlled for energy intake. Mean energy (kcal) from baseline, modelled control and modelled intervention were compared to estimated energy needs using paired samples t-tests. Individuals included in the present analysis were assigned EFSA DRVs for critical nutrients depending on sex and age. Each individual’s mean daily micronutrient intakes were compared to their corresponding EFSA DRVs (PRIs or AIs).

## Results

### Environmental indicators

The presented analysis was based on 20 baseline assessments from MyPlanetDiet, evenly represented by sex and age group. The participants had a mean age of 40 years and were representative of the total MyPlanetDiet RCT (Supplemental Table 3 Online Resource 1). Diet-related GHGE were highest among baseline diets (5.5 ± 0.4 kg CO_2_ equivalents per day) (*p* = 0.006) (Table 4). Modelled control diets had a mean diet-related GHGE of 5.4 ± 0.3 kg CO_2_ equivalents/day, 3% lower than baseline diets. GHGE associated with modelled intervention diets was 15% lower relative to baseline with mean daily GHGE of 4.7 ± 0.3 kg CO_2_ equivalents. There were significant differences in energy intake across baseline, modelled control, and modelled intervention diets (Table 5) with modelled intervention diets having the highest energy due to the energy-tiered feedback structure (*p* < 0.001). When GHGE were adjusted for energy intake, there were larger decreases in GHGE in both modelled control (-7%) and modelled intervention (-34%) diets relative baseline (*p* < 0.001). Baseline diets remained the highest for diet-related GHGE when adjusted for energy (2500 kcal) emitting 7.1 ± 0.5 kg CO_2_ equivalents on average per day. Modelled intervention diets’ mean GHGE/2500 kcal was 29% lower than the control diets. Baseline diets were adjusted based an individual’s respective energy needs resulting in mean GHGE of 7.6 ± 0.7 kg CO_2_ equivalents, 38% higher than unadjusted baseline intakes (Online Resource 1). Total water footprint (litres per day) was higher in modelled control diets relative to baseline (+ 5%) but when adjusted for energy (2500 kcal) the control diets had 4% lower water footprint (*p* < 0.001). The modelled intervention diets had the lowest mean water footprint in both total water footprint (838.7 ± 99.2 L of water/day) and water footprint per 2500 kcal (873.2 ± 118.7). The modelled intervention diets had 23% lower mean water footprint per 2500 kcal relative to baseline and 20% lower relative to the modelled control (*p* < 0.001). In baseline diets adjusted for energy needs, the mean water footprint was 31% higher than baseline diets as reported by participants (Online Resource 1).

**Table 4:** Diet-related greenhouse gas emissions and water footprint of baseline, modelled control, and modelled intervention diets

	Baseline (*n* = 20)			Control (*n* = 20)			Intervention (*n* = 20)			
	Mean		SE	Mean	SE	% change	Mean	SE	% change	*p*-value^a^
GHGE	5.5	0.4		5.4	0.3	-3.1	4.7	0.3	-14.6	0.006
GHGE/2500 kcal	7.1	0.5		6.6	0.3	-7.0	4.7	0.2	-33.8	< 0.001
Water footprint	862.3	124.6		902.3	122.2	+ 4.6	838.7	99.2	-2.7	0.264
Water footprint/2500 kcal	1139.3	150.7		1089.2	132.9	-4.4	873.2	118.7	-23.4	< 0.001

SE: standard error of the mean; GHGE: Mean daily greenhouse gas emissions as kilograms carbon dioxide equivalents; Water footprint, Mean daily water footprint in litres of water

^a^: All absolute values were adjusted for differences in energy intake and analysed for significance using repeated measure one-way ANOVA. All significant values remained significant in the adjusted analysis

### Nutrient intakes

**Fig. 2:**
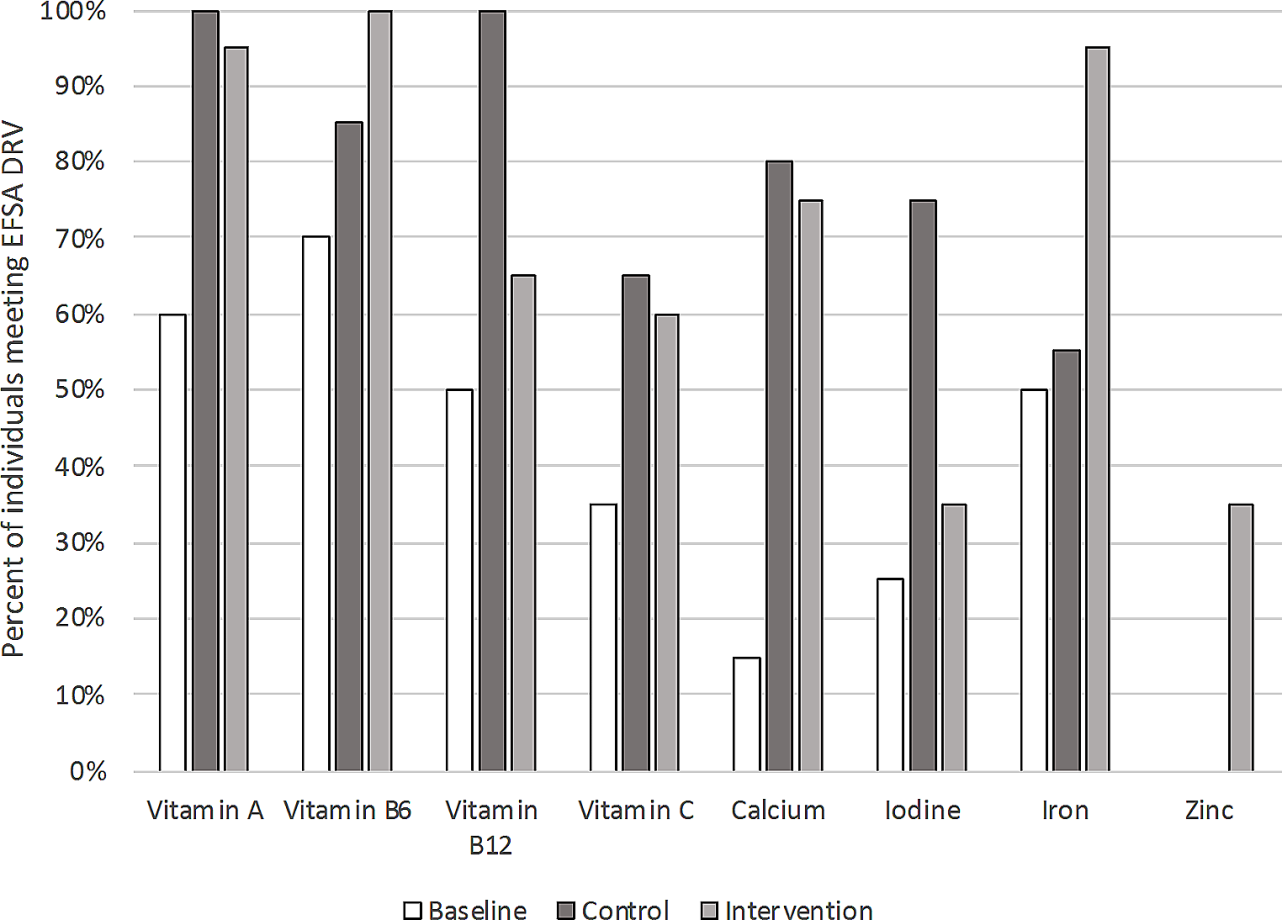
Percent of individual diets meeting EFSA DRV (PRI or AI); EFSA, European Food Safety Authority; DRV, dietary reference value; PRI, population reference intake; Vitamin D, Vitamin B12 and Iodine; EFSA adequate intake (no PRI available); Zinc requirements use highest phytate recommendations for both males and females

Nutrient intakes were significantly different between baseline, modelled control, and modelled intervention diets (*p* < 0.05) (Table 5). Energy intakes were highest in the modelled intervention diets (2562.5 ± 112.9 kcal/day) compared to baseline (1975.4 ± 100.1 kcal/day) and control (2088.3 ± 341.5 kcal/day). Mean energy from the modelled control diets and baseline diets were significantly different to mean estimated energy needs (*p* < 0.001) (Online Resource 1). The modelled intervention diets had highest percentage of energy from carbohydrates and the lowest percentage of energy from fat and saturated fats. Percent energy from protein was highest in the modelled control diets, while protein intake relative to body weight was the same in modelled control and modelled intervention diets and higher than baseline. Intakes of calcium, iodine, vitamin C and vitamin B12 were highest in modelled control diets. Fibre, iron, zinc, folate, vitamin A and sodium were highest in the modelled intervention diets. Intakes of critical micronutrients increased in both modelled diets relative to participants’ baseline diets.(Fig. 2)

**Table 5:** Mean baseline nutrient intakes compared to mean nutrient intakes of modelled control and intervention diets

	Baseline (*n* = 20)		Control (*n* = 20)		Intervention (*n* = 20)		
	Mean	SE	Mean	SE	Mean	SE	*p*-value^a^
Energy (kcal)	1975.4	100.1	2088.3	341.5	2562.5	112.9	< 0.001
Carbohydrate (%)	41.1	1.7	43.0	1.2	49.2	1.0	< 0.001
Fibre (g)	18.2	1.1	24.7	0.9	49.1	1.9	< 0.001
Fat (%)	37.2	1.2	33.3	0.9	31.6	0.8	< 0.001
Saturated fat (%)	13.8	0.5	12.1	0.4	10.1	0.4	< 0.001
PUFA (%)	5.7	0.3	5.6	0.2	6.5	0.2	< 0.001
MUFA (%)	13.0	0.6	11.6	0.5	11.6	0.4	0.002
Protein (%)	17.5	1.1	19.8	0.9	16.4	0.4	< 0.001
Protein (g/kg)	1.1	0.9	1.3	0.9	1.3	0.4	< 0.001
Sodium (mg)	2537.9	229.3	2363.2	175.7	2856.2	210.4	0.006
Calcium (mg)	702.3	60.2	1141.7	43.4	1084.5	43.4	< 0.001
Iron (mg)	11.5	0.7	12.5	0.6	20.2	0.8	< 0.001
Zinc (mg)	8.3	0.6	10.1	0.4	13.6	0.6	< 0.001
Iodine (µg)	125.8	13.9	195.6	14.4	133.3	8.6	< 0.001
Vitamin B6 (mg)	2.1	0.3	2.5	0.3	2.6	0.2	< 0.001
Folate (DFE) (µg)	268.7	25.3	382.7	25.1	446.7	24.8	< 0.001
Vitamin B12 (µg)	4.9	0.8	6.4	0.7	4.9	0.4	< 0.001
Vitamin A (mg RE)	977.6	149.7	1197.5	95.0	1845.6	118.5	< 0.001
Vitamin C (mg)	95.4	16.1	142.7	12.7	136.6	13.3	< 0.001

SE: standard error of the mean; Significance measured with repeated measure one-way ANOVA; *p* < 0.05 was considered statistically significant; PUFA, polyunsaturated fatty acids; MUFA: monounsaturated fatty acids; DFE: dietary folate equivalents; RE, retinol equivalents

^a^: All absolute values were adjusted for differences in energy intake and analysed for significance using repeated measure one-way ANOVA. All values remained significant in the adjusted analysis

### Nutrient intakes relative to EFSA recommendations

A greater proportion of modelled control diets and modelled intervention diets fell within dietary reference value (DRV) relative to baseline diets [38]. Modelled control diets had the highest proportion of individuals above the DRV for vitamin A, vitamin B12, vitamin C, calcium, and iodine [38]. Modelled intervention diets had the highest proportion above the DRV for vitamin B6, iron and zinc [38]. No baseline or modelled diet met the EFSA adequate intake (AI) for vitamin D (15 µg/day) [38]. Baseline diets and modelled control diets were below the respective DRV for zinc. The present DRV used for zinc is based on the highest level of phytate intake, 12.7 mg/day for females and 16.3 mg/day for males [38]. In the modelled intervention diets, 35% of diets were above the DRV for zinc [38].

## Discussion

This paper describes the protocol of a dietary intervention study and the development and testing of personalised nutrition feedback for a more sustainable and healthy diet. The personalised feedback was designed to be nutritionally adequate and acceptable while decreasing diet-related GHGE. If demonstrated to be effective, use of the presented personalised nutrition feedback can be easily scalable due to the reproducibility of the method [20]. Dietary advice was tailored to an individual based on their baseline dietary intake and their nutrition needs. Each individual received actionable feedback messages for critical foods groups with specific intake recommendations on a daily (e.g. one serving per day of dark green vegetables) or weekly (e.g. have oily fish once per week) basis. To our knowledge, the work presented here is the first to describe the development of a standardised approach for providing personalised healthy and sustainable dietary advice to individuals. Several other research groups have published reference diets for sustainable healthy eating, but have used one energy tier for recommendations, such as 2500 kcal or 10 MJ per day [3, 6, 7]. Such reference diets along with consideration of national dietary intakes were used as a basis for the present work, which was then expanded to consider how personalised factors influence nutrition needs and necessary dietary changes.

The dietary feedback framework was tested using a small sample of the MyPlanetDiet RCT to assess the effectiveness and impact of the personalised nutrition feedback on environmental and nutrient intake outcome measures. Diets modelled to the intervention recommendations had significantly lower GHGE relative to both baseline diets and modelled control diets. Energy intake in the modelled intervention diets were significantly higher due to the energy tiered feedback system which uses an individual’s estimated energy needs to provide dietary feedback. When each group’s diet-related GHGE were adjusted for energy intake (GHGE per 2500 kcal), larger differences in diet-related GHGE relative to baseline (-34%) were observed. Previously published research which modelled the environmental impacts of dietary changes found diet-related GHGE were significantly reduced (20–50%) with more plant-based diets [4, 27, 39, 40]. Our initial analysis aligns with previous research groups’ findings and with the primary outcome of MyPlanetDiet (20% decrease in GHGE). However, despite significant reductions in GHGE, the mean diet-related GHGE of the modelled intervention diets is still 2.3–2.7 kg CO_2_-eq above the recommended GHGE limit from previous reference diets [7, 14]. Due to the inclusion criteria of MyPlanetDiet, participants were screened and included if they followed key behaviours of moderate to high emitting diets. MyPlanetDiet was designed to be acceptable to those who consume such diets by considering dietary preferences (i.e. distribution of meat intake or portion sizes) from nationally representative dietary intake data. This consideration may impact the scope of reducing environmental impact.

Diet-related GHGE were marginally lower in the modelled control diets (-3% compared to baseline), but higher than previous modelled research which has estimated reductions in diet-related GHGE by 13% with adherence to food-based dietary guidelines [5]. However, while all participants in MyPlanetDiet were told to limit their intakes of discretionary foods, this was not a personalised feedback message in the study and was not modelled in the present work. Reducing discretionary foods in the diet is likely to reduce energy intakes, GHGE and water footprint and improve energy balance. Research suggests that eating within an energy balance is a core component for making a diet more sustainable and healthier [5].

The results of the modelled data suggest that adherence to both control and intervention diet recommendations would lead to improved nutrient intakes. Only the modelled intervention diets had mean values for percent total energy from carbohydrates, fat, and protein and total grams fibre within EFSA DRV macronutrient ranges [38]. The modelled intervention had the lowest percent energy saturated fat (10%) and was closest to EFSA reference value of < 10% [38]. Mean macronutrient distributions of the modelled intervention diets were comparable to other reference diets [6, 7]. Micronutrient intakes were significantly higher in both control and intervention diets relative to baseline. The intervention diets had mean micronutrient intakes above EFSA DRVs apart from iodine and vitamin D [38]. Control diets had mean micronutrient intakes above EFSA DRVs except for vitamin D and zinc [38]. However, no diet (baseline, modelled control, or modelled intervention) was nutritionally adequate for all nutrients for all individuals based on EFSA DRVs. Previous research has concluded that sustainable diets could lack sufficient intakes of vitamin D, vitamin B12 and iodine [6, 10, 13]. These findings may be indicative of the need for more animal sourced foods in the diet, increases in fortification of plant-based foods or supplementation. It is worth noting that baseline diets had fewer individual diets meeting adequate intakes for iodine and vitamin B12 relative to the modelled intervention diets, despite having more animal sourced foods. Yet contributions to critical micronutrients in the modelled intervention diets were predominantly from plant-based sources, and previous research has linked such diet patterns with increased risk of nutrient inadequacies due to reduced bioavailability [10, 41–45]. Future research should consider the difference in animal- and plant-based contributions to nutrient intakes and the corresponding impacts to health and nutrition status.

The approach used to model diets was consistent for each individual. For example, carrots were added into the diets of each individual who were not yet meeting their recommendation for red/orange vegetables in the intervention diet. Carrots are rich in beta-carotene and contribute to retinol equivalents (vitamin A), while other red vegetables such as red bell peppers would be higher in vitamin C, a micronutrient which only 60% of modelled intervention diets were above the EFSA PRI as opposed to 95% for vitamin A. Similarly, changes were made to daily diets based on ascending food code. This helped to eliminate researcher bias in the diet modelling, but also may have decreased diet variability across the sample. For example, many individuals had eggs, poultry, and pork in the same day, but eggs have a lower food code in Foodbook24. Eggs therefore remained in the diet over poultry or pork in these cases affecting the nutrient intake results. However, it is important to note that the findings of the decision tree testing are based on systematic decisions designed to replicate adherence to the personalised nutrition feedback. Individuals will respond to dietary advice differently in real life, which will be examined in future analysis from MyPlanetDiet. The variability in dietary intakes will be assessed using the study’s dietary assessments, and participants’ perceptions of the dietary advice will be captured in the diet-change tolerability questionnaire. The analysis of dietary changes over time and the acceptability of such changes are two outcomes that will be reported in future work.

### Strengths and limitations

The MyPlanetDiet RCT is the first of its kind with the primary outcome of reducing environmental impact through personalised dietary advice at an individual level. To our knowledge, the study will be the first to test adherence to a more sustainable diet and to assess the safety and nutrition adequacy of such a diet in a human intervention study. Biological samples collected during the study will be used to assess markers of metabolic health, micronutrient status, amino acid status and gut microbiota composition. Participants on the study also completed tolerability questionnaires to determine the acceptability of the dietary change recommendations.

The nutrition feedback provided in the study has been refined, analysed, and tested to minimise risk of nutrient inadequacies. The decision trees have been tested using a modelled, personalised approach with a robust method designed to reduce bias. In this modelled approach, foods were exclusively modelled based on feedback participants received, then based on daily intake by ascending food code. While this kept the diet modelling consistent for each individual, it may skew nutrition data due to reduced diversity within critical food groups which impacts micronutrient intakes as described above in relation to red/orange vegetables. Similarly, a limitation of the dietary assessments is that the data is based on what individuals chose to report at one point in time. Further context to dietary assessment such as broad history of energy intakes, micronutrient intakes, or weight history could not be considered in the present analysis. Lastly, the only modelled changes that were made to individual diets were the changes specifically advised to each individual in their personalised nutrition feedback. Discretionary foods and other foods outside the feedback’s target food groups were unchanged. While we can anticipate individuals will make other dietary changes to compensate for the recommended dietary changes, these are unable to be captured or estimated in this type of analysis. The results of the MyPlanetDiet study will show the reality of dietary changes individuals made as part of the RCT’s dietary assessments.

## Conclusions and future perspectives

The methods and study described will fill a critical gap in understanding the effectiveness, nutritional adequacy, safety, and acceptability of more sustainable dietary advice. Based on modelled data presented here, it is anticipated that the recommended dietary changes of both the intervention and control groups will decrease environmental impact and improve nutrient intakes. However, as we transition to a more sustainable healthy diet it is important to consider the potential impact that dietary change will have on the food system. For example, compared to current intakes, sustainable diets may increase fish consumption, so it is important to develop alternative practices to deliver a resilient fish supply without overfishing open waters. Future MyPlanetDiet results will help to better understand how individuals change their diets in response to sustainable dietary advice. This perspective should then be considered as part of a food systems approach alongside other system-level changes such as ethical and sustainable food production and processing as we move to protect our environment. Similarly, future results will report diet-related GHGE and water footprint of diets pre- and post-intervention, but modelled data shows that the diet recommendations are effective in reducing GHGE in line with the study’s primary outcome and power calculation. While previous research and the current analysis shows that a more sustainable diet would not be nutritionally adequate, modelled data shows macro- and micronutrient intakes will improve. Nutrition status will be tested with biological data collected in the MyPlanetDiet intervention. It is unclear how participants will alter their diets to compensate for the recommended dietary changes, but these results will be reported as part of the MyPlanetDiet study.

## Electronic supplementary material

Below is the link to the electronic supplementary material.

Supplementary Material 1

## Data Availability

Full protocol and data may be available upon request subject to ethical restrictions.

## Appendix a consent form and related documentation

**Table:**

INFORMED CONSENT FORM: Please initial each section to show you have read and understood each statement. If you agree to take part, please sign the bottom of the form.			
1.	I confirm that I have read, or had read to me, and understand the information leaflet about this study dated 24/MAY/2021, version 1.0 and that I have been given a copy to keep.		
2.	I have had the opportunity to ask questions, and these have been answered fully. I understand the purpose of the research.		
3.	I understand that my participation is voluntary, and I am free to withdraw at any time, without giving any reason and without my legal rights being affected. In the event that I withdraw from the study, I agree that the coded personal data collected prior to withdrawal may still be processed along with other data collected as part of the study to preserve the integrity of the study.		
4.	I understand the study is being conducted by researchers from << SITE>> and that my personal information will be held securely on university premises and handled in accordance with the provisions of the General Data Protection Regulation (GDPR) (EU) 2016/679 / Data Protection Act 2018.		
5.	I understand that data collected as part of this study may be looked at by authorised individuals from << SITE>> and regulatory authorities where it is relevant to my taking part in this research. I give permission for these individuals to have access to this information.		
6.	I understand that the sponsors and investigators have the necessary insurances as required by law to conduct this study.		
7.	I understand that no information will be divulged and the results of any tests involving myself will never reveal my identity. I give permission for all information collected about me to be stored for possible future research on diet and health without my further consent being required, but subject to ethics approval.		
8.	I agree for my blood and urine samples to be stored for further analysis in this research which may involve sending samples to 3rd party laboratories for analysis. This does not change the conditions of what I am agreeing to in this consent form. The remaining sample (if all the sample is not used in the analysis) with my permission will be placed in long term storage in the biorepository and may be used for related research in the future. This research would be subject to ethics approval.		
9.	I agree that the study team can keep in contact with me about this study and approach me directly about assessments.		
10.	I agree to take part in the above study.		
11.	I have received a copy of this document for my records.		

__________________________ ________________________ _________.

Name of Participant (please print) SignatureDate (DD/MON/YYYY).

_________________________ _________________________ _________.

Name of Investigator Signature Date (DD/MON/YYYY).

**SuHeGuide Participant Information Leaflet**.

You are being invited to take part in this study which aims to improve dietary guidelines using a personalised approach. This study is conducted by << PI NAME>> and << MPD RESEARCHER/PHD CANDIDATE>> in << SITE>> and is funded by the Department of Agriculture, Food & the Marine.

**Introduction**.

Before you decide if you want to take part, it is important that you understand why the research is being done and what it will involve. This process is known as informed consent. This leaflet will give you detailed information about the research study. Please take the time to read it carefully and discuss it with others if you wish. Please ask us if there is anything that is not clear or if you would like more information. When you are sure that you understand this study, and would like to take part, you will be asked to sign a consent form.

**What is this research about?**

The way we produce and eat food has an impact on our health and on the environment. Research suggests that we will need to make some different choices when it comes to buying and eating food in order to reduce the pressure this is putting on our environment. However, there are still some questions that we need to answer about the best dietary habits to protect both human and planetary health. This study is looking at ways to develop dietary guidelines that will help to protect and promote health as well as reduce greenhouse gas emissions, which is one of the measurements we use to assess environmental damage.

**Why am I doing this research?**

We are conducting this study to test if it is possible to design a healthy diet which is better for you and at the same time better for the environment. We would like to explore whether a healthy diet that is also more climate-friendly is acceptable to a person like you. Our tests of this diet will also question whether it is capable of providing you with all of the nutrients that you need, in comparison to a healthy diet that does not take environmental considerations into account. To do this, we will collect information on your dietary intake, analyse nutrient levels in your urine and blood, and take measurements such as your weight. The information collected in this study will be used to help develop dietary guidelines that take account of the impact of food on the environment. We will share the outcomes of our studies with the Government and policy makers.

**Why have you been invited to take part?**

We are seeking healthy adults aged 18 – 64 years to participate in this study.

**How will your data be used?**

By participating in this study, your information (also called “personal data”) will be collected for the purposes outlined in this Participant Information Leaflet. We will collect information using some paper-based questionnaires and some online questionnaires.

Personal data collected from you during this study and the results of the study may be presented for scientific purposes. However, you will never be identified individually during these presentations or in any reports or publications. To ensure confidentiality, we will replace your name with a study code called a Participant Number. Data that directly identifies you will be stored in a locked filing cabinet, separate to all the other study documentation. Only study personnel can match your name to your unique Participant Number.

All information collected in this study will be entered into a secure electronic database, so that it can be statistically analysed. Any information that could identify you **will not be stored** in the database.

The blood and urine samples you provide will be stored and tested at a later stage for markers of nutritional status and health. Any additional blood or urine that is not used as part of this testing, with your permission, will be placed in long term storage (biorepository). These samples may be used in subsequent related research, but this research would be subject to ethics approval.

This study is being undertaken as part of an educational programme, where this work will contribute to the student researchers receiving their doctorate qualification. The anonymised results of this study will be presented as part of the student’s final thesis.

**What will happen if you decide to take part in this research study?**

If you decide to take part, you will be asked to attend our study site in the << SITE>> two times, 12 weeks apart, and have at least four phone calls over the 12-week period.

Initially, you will complete a screening questionnaire and your eligibility will be checked by the research team. You will be asked about your age, medical conditions, and the food you eat during screening.

If you are suitable, you will have a phone call with the researcher who will introduce the study and obtain informed consent. You will answer questions on your demographics, lifestyle, medical history, and use of medications and supplements. You will be randomly assigned to follow a diet that is designed for you based on your current diet and healthy eating guidelines that either includes or does not include environmental considerations. Neither you nor the researcher will be able to choose which diet you are assigned, this is randomly assigned by a computer.

At home, you will complete questionnaires on your diet at three timepoints using a simple, user-friendly online tool. This will include two types of reporting. Firstly, on three different days, you will be asked to record your diet over the previous 24 h. Secondly, you will be asked to answer questions on your diet, looking over the previous month. These assessments will take approximately 15 min each.

The onsite appointments will last approximately 60 min. All visits will be conducted according to public health COVID recommendations. You will be asked to attend the site having fasted (no food or drinks except water) since 22:00 the night before. One these days, the following assessments will take place:

- We will take your body measurements, like height and weight and measure your blood pressure.

- You will complete questionnaires related to physical activity, diet, and food waste.

- You will be asked to provide a urine sample and a blood sample (6 teaspoons) will be collected by a trained and experienced nurse. These samples will be frozen and analysed for nutrients and other health related biomarkers at a later date.

- Finally, a nutritionist will explain the changes required to your current diet for the next 12 weeks. The changes will be personalised to you, based on the foods you currently eat. You will receive a report that summarises the changes you are expected to adhere to.

The researcher will send a weekly email/SMS and schedule a phone call with you every three weeks to ensure you are getting on ok. The phone calls will take approximately 10 min but may last longer if you need. You can contact the team directly at any time, contact details are at the bottom of this leaflet.

**How will your privacy be protected?**

Any personal data which you provide to the University will be treated with the highest standards of security and confidentiality, in accordance with Irish and European Data Protection legislation. Signing the Informed Consent Form means that your personal data will be used for the purposes outlined in this Participant Information Leaflet. Documentation will be stored securely in locked cabinets or password protected files, and access will be restricted to the research team. We will retrain anonymous coded data records and any remaining urine and blood samples for 10 years at which point they will be safely destroyed. If you wish to withdraw from the study your identity and contact details will be removed immediately.

**What are the benefits of taking part in this research study?**

If you decide to take part, you will be contributing to research which is aiming to improve dietary guidelines for the general population. You will get feedback on your existing diet and will benefit from tailored nutritional advice from a qualified nutritionist. You will have nutritionist support at any stage in the trial and information on your diet and recommended changes to make will be provided to you in a comprehensive report. You will be provided with personalised resources and recipes to help you make the recommended changes.

**What are the risks of taking part in this research study?**

There are no risks associated with following the diets in this study. The guidelines have been put together to ensure that you meet all of your nutritional needs. If you do not follow these guidelines, there is a risk that you may under consume or over consume some nutrients. There are some small risks related to blood collection. These include pain from the needle going through your skin, bruising, light-headedness, possible fainting, and rarely, infection. However, the risks will be minimal. The blood taking is routine and a fully trained individual will take the blood samples to ensure that any discomfort is kept to a minimum.

**Can you change your mind at any stage and withdraw from the study?**

Your decision to take part in this study is entirely voluntary. You will receive a copy of this leaflet so that you know what is expected of you at any time. You may end your participation in this study at any time simply by notifying the researcher that you have been in touch with. You can find their details at the end of this leaflet. You do not have to give a reason for withdrawing.

If you decide to withdraw from the study earlier than planned, the research team may ask you some questions about being in the study. The information collected and the biological samples stored, from the day you have signed the consent form and up to the last study visit, will be used for statistical analysis unless you also withdraw your consent for them to be used in this way. If you withdraw your consent, none of your data will be used. Following your withdrawal, no new data will be collected.

**How will you find out what happens with this project?**

On completion of the project, after all participants have finished and the results are known, the researchers will put together a summary of the study results. With your permission, we will send a copy of the results summary to you. The results of the study will be published in scientific papers and will be publicised.

**Will I be reimbursed for travel expenses?**

Yes, reasonable travel expenses will be reimbursed. Please discuss this with the researcher before travel and keep all travel receipts, otherwise cost cannot be reimbursed.

**Contact details for further information**.

If you have any additional questions or would like to hear more about this study, please contact the research team at XXXXXXXXXXXXXX@XXXXX.XX.

**Thank you for your interest in this study and for taking the time to read through this information leaflet.**

## Appendix B: Biological samples and clinical measurements

**Biological samples**.

Participants were asked to collect a midstream first void urine sample at home, place the sample on ice in a thermos bag and bring it to the test centre on the morning of both visits. A sub-group of participants were asked to collect a faecal sample in the 48 h prior to both visits. Fasted blood samples were collected into EDTA (6 mL) and serum tubes (12 mL) by a phlebotomist. Serum tubes were kept at room temperature, in the dark, for at least 30 min before processing. To stabilise the vitamin C in EDTA, 400 µl of 10% metaphosphoric acid (MPA) was added to two 400 µl aliquots of EDTA. All biological samples were processed as require, aliquoted and stored in -80 °C freezer until analysis.

Laboratory analysis will include biomarkers of nutritional status including vitamins C, E, D, E, K1, iron stores, iodine, and carotenoids. Amino acid status and markers of metabolic health including lipids, glucose, insulin, CRP, and interleukins will be analysed. Faecal samples will be used to analyse gut microbiota composition.

**Clinical measurements**.

Height was measured without shoes to the nearest millimetre using a freestanding Leicester stadiometer (Seca, Birmingham, UK). Fasting weight and body composition were measured using a Tanita body composition analyser BC-420MA (Tanita Ltd., Manchester, UK). Waist and hip circumference were measured with participants standing with arms down. Anthropometric measurements were taken in duplicate at visit 1 and visit 2 (Table 1) and each mean was used in final analysis. Blood pressure was measured on the participants non-dominant arm using an Omron M6 Comfort HEM-7360-E (Omron Healthcare Ltd., Brighton, UK) in a seated position, feet on the floor and arm resting on a flat surface after the participant had been resting for at least 10 min. Two blood pressure measurements were taken unless the difference in systolic or diastolic was greater than 5mmHg. If there was a larger than 5mmHg difference, a third measurement was taken, and the mean was used for data analysis. If an individual’s blood pressure was >140/90mmHg and was not currently prescribed blood pressure lowering medications, they were excluded from the study at that time.
